# The Long-Term Clinical and Economic Impact of Universal Varicella Vaccination in Slovenia

**DOI:** 10.36469/001c.37308

**Published:** 2022-09-20

**Authors:** Colleen Burgess, Stephanie Kujawski, Ajda Lapornik, Goran Bencina, Manjiri Pawaskar

**Affiliations:** 1 Merck & Co., Inc., Rahway, New Jersey; 2 MSD Inovativna zdravilla d.o.o., Ljubljana, Slovenia; 3 MSD, Madrid, Spain

**Keywords:** varicella vaccination, vaccination strategy, Slovenia, cost effectiveness, health economics

## Abstract

**Background:** Despite the substantial burden of varicella infection, Slovenia does not currently have a universal varicella vaccination (UVV) program. We modeled the long-term clinical and economic impact of implementing 2-dose UVV strategies compared with no vaccination in Slovenia. **Methods:** A previously published dynamic transmission model was adapted to the demographics, varicella seroprevalence, herpes zoster incidence, and contact patterns in Slovenia. Six 2-dose UVV strategies, vs no vaccination, were considered over a 50-year period, including monovalent vaccination (Varivax^®^ [V-MSD] or Varilrix^®^ [V-GSK]) at ages 12 and 24 months, or monovalent vaccination at 15 months followed by monovalent or quadrivalent vaccination (ProQuad^®^ [MMRV-MSD] or Priorix- Tetra^®^ [MMRV-GSK]) at 5.5 years. Costs, quality-adjusted life-years, and incremental cost-effectiveness ratios vs no vaccination were calculated to assess the economic impact of each strategy from payer and societal perspectives. **Results:** The incidence of varicella infection was estimated as 1228 per 100 000 population in the absence of UVV. Over 50 years, depending on vaccination strategy, UVV reduced varicella cases by 77% to 85% and was associated with substantial reductions in varicella deaths (39%-44%), outpatient cases (74%-82%), and hospitalizations (74%-82%). The greatest reductions were predicted with V-MSD (15 months/5.5 years) and V MSD/MMRV-MSD (15 months/5.5 years). **Discussion:** All 2-dose UVV strategies were cost-effective compared with no vaccination from payer and societal perspectives, with V-MSD (15 months/5.5 years) being the most favorable from both perspectives. **Conclusion:** Policymakers should consider implementing UVV to reduce the burden of varicella disease in Slovenia.

## BACKGROUND

Varicella is a highly contagious disease caused by the varicella zoster virus and is one of the most common vaccine-preventable childhood diseases.^1,2^ Varicella infection is characterized by a generalized pruritic vesicular rash. While usually mild, varicella can result in severe complications such as bacterial infections, pneumonia, cerebellitis, and encephalitis.^2,3^ Neonates, immunocompromised individuals, and adults are more likely to experience complications; in rare cases, infection can lead to death.^4^

Although preventable, varicella incidence remains high in the absence of universal varicella vaccination (UVV) in many European countries.^5^ Without UVV, the annual incidence of new cases across Europe is estimated to be 5.5 million, with 3 million occurring in children less than 5 years of age.^5^ An estimated 3 million to 3.9 million individuals are expected to have varicella-related outpatient visits annually, with 18 200 to 23 500 hospitalized.^5^ In Slovenia, where no UVV program currently exists, the annual incidence of reported cases was 467 per 100 000 population in 2018, with young children and adults most impacted; however, due to underreporting, this is likely to be an underestimation of the true burden of disease.^6^ Between 1996 and 2005, the hospitalization rate in Slovenia ranged from 3.3 per 1000 varicella cases in children aged 10 to 14 years to 23.3 per 1000 varicella cases in adults 30 years of age and older.^7^ Among infants under 1 year of age, 19.5 per 1000 varicella cases resulted in hospitalization in Slovenia.^7^ Moreover, the economic burden of varicella disease can have a substantial societal impact, largely due to indirect costs resulting from workdays missed by adults or by parents or caregivers of children who had varicella infection.^8^

Countries that have implemented childhood UVV have observed significant declines in varicella disease.^9–15^ Randomized controlled trials have reported high vaccine efficacy for 2 doses of varicella vaccine (95.4%-98.3%), and long-term follow-up has demonstrated vaccine effectiveness of 90%.^16–18^ Importantly, vaccination has been associated with declines in varicella-related mortality and morbidity, thereby reducing the economic and societal burden of varicella infection.^15,19^ Routine childhood vaccination for varicella has been included in national immunization programs in 42 countries worldwide, although policies vary by country.^20^ However, currently there are no recommendations for UVV in Slovenia; recommendations are only for children with high-risk conditions.^21,22^

This study models the long-term clinical and economic impact of implementing 2-dose UVV strategies vs no vaccination in Slovenia from payer and societal perspectives.

## METHODS

### Model Overview

A previously published dynamic transmission model of varicella infection^23^ was adapted to the demographics, varicella seroprevalence, herpes zoster incidence, and social contact patterns in Slovenia and used Slovenia-specific inputs for resource use, unit costs, and workdays lost for indirect costs. Where Slovenia-specific data were not available, proxy estimates from other countries were used. Key model parameters, inputs, and data sources are summarized in **Supplementary Tables S1-S4**. Model equations are provided in Wolfson et al.^23^

### Dynamic Transmission Model

The model used country-specific data from the United Nations for age-specific population size, all-cause mortality rates, and fertility rates.^24^ The epidemiological model simulated the transmission of varicella infection in Slovenia, based on an MSEIR (maternally protected, susceptible, exposed, infected, and recovered) structure as previously described by Wolfson et al^23^ and used Slovenia-specific social contact patterns.^25^ Transmission rates based on age-specific mixing were specific to Slovenia and were determined during model calibration (details in Wolfson et al^23^). The parameters of the epidemiological model used age-specific case fatality ratios for varicella, regardless of treatment setting and varicella case type, which are incremental to background mortality.^23^ The disease-specific model parameters are listed in **Supplementary Table S2**.

The model supported detailed input of both direct (healthcare utilization costs associated with varicella infection) and indirect costs (workdays lost by patients and parents or caregivers for cases in children <18 years old). Where available, Slovenian data, sourced from the literature and public records, were used for healthcare resource utilization, unit costs, and workdays lost (**Supplementary Table S3**). Proxy data from other countries were used when Slovenian data were unavailable.

The economic model combined the change in health utility from perfect health with estimated life-years lost due to varicella to produce quality-adjusted life-years (QALYs). The model inputs included health utility values for natural varicella and breakthrough varicella by age (**Supplementary Table S4**) as adjustment factors, applied multiplicatively to healthy utility values.^23,26,27^ Cost outcomes were modeled from payer and societal perspectives and costs and QALYs were discounted at 3% annually.^28^ Cost inputs were in 2020 euros (€) (**Supplementary Table S3**).

The vaccine characteristics and parameters for the modeled varicella vaccines are detailed in **Supplementary Tables S1 and S2**. Vaccine prices were sourced from list prices, and price parity was assumed between both varicella vaccines and both measles, mumps, rubella, and varicella (MMRV) vaccines (Merck & Co, Inc [MSD] vs GlaxoSmithKline [GSK]; **Supplementary Table S3**).^29–31^

### Model Outcomes

The dynamic transmission model estimated the following key outcomes (annually), modeled over a 50-year time horizon: (1) varicella incidence (natural and breakthrough varicella); (2) varicella cases and deaths; (3) number of QALYs lost; (4) health resource utilization; and (5) incremental cost-effectiveness ratios (ICERs).

The model was run using Mathematica v12.1 (Wolfram Research, Champaign, Illinois). Costs, resource use, and QALYs lost were calculated from the health outcomes in the dynamic transmission model at each time step.^28^ The cost-effectiveness of varicella vaccination strategies (**Table 1**) was then assessed using cumulative health and cost outcomes, and compared with the no-vaccination strategy. Strategies were considered cost-effective at the ICER threshold for Slovenia of €25000/QALY.^32^

**Table 1.** Varicella Vaccination Strategies Considered

**Strategy**	**First Dose**		**Second Dose**	
	**Vaccine**	**Age**	**Vaccine**	**Age**
A (short-interval)	V-MSD	12 mo	V-MSD	24 mo
B (short-interval)	V-GSK	12 mo	V-GSK	24 mo
C (medium-interval)	V-MSD	15 mo	V-MSD	5.5.y
D (medium-interval)	V-GSK	15 mo	V-GSK	5.5 y
E (medium-interval)	V-MSD	15 mo	MMRV- MSD	5.5 y
F (medium-interval)	V-GSK	15 mo	MMRV- GSK	5.5 y

All vaccination strategies included a single-dose catch-up at age 3 to 5 years in the first year of the varicella program.

Abbreviations: GSK, GlaxoSmithKline Biologicals, Belgium, UK; MMRV, measles, mumps, rubella, and varicella vaccine; MSD, Merck & Co, Inc, Rahway, New Jersey; V, varicella vaccine.

### Vaccination Strategies

Current UVV programs typically comprise a 2-dose regimen; therefore, a total of 6 two-dose vaccination strategies, compared with no vaccination, were considered over a 50-year time horizon postvaccination, including short-interval (12 months/24 months) and medium-interval (15 months/5.5 years) strategies for Varivax® (V-MSD, Merck & Co, Inc, Rahway, New Jersey), ProQuad® (MMRV-MSD, Merck & Co, Inc, Rahway, New Jersey), Varilrix® (V-GSK, GlaxoSmithKline Biologicals, Belgium, UK), and PriorixTetra® (MMRV-GSK, GlaxoSmithKline Biologicals, Belgium, UK) (**Table 1**). For the medium-interval strategy, ages for vaccine administration aligned with the existing measles, mumps, and rubella (MMR) vaccination schedule in Slovenia. The MMR vaccine is administered between 11 and 18 months of age (first dose) and between 5 and 6 years of age (second dose).^33^ In all varicella vaccinations strategies, the first dose was assumed to be monovalent and the second dose either monovalent or quadrivalent. In line with current MMR vaccination coverage rates in Slovenia, vaccination coverage was assumed to be 90% for both varicella doses, with a single dose of catchup vaccination at age 3 to 5 years (90% coverage) occurring in the first year of the varicella program.^34^

### Calibration

The model was calibrated to fit observed varicella seroprevalence and herpes zoster incidence by age in Slovenia.^35,36^ The calibration process involved adjusting contact rates based on a Slovenia-specific contact matrix.^25^ Contact rates were then used to calculate the transmission matrix and independently calibrated to herpes zoster incidence by adjusting herpes zoster reactivation rates. Results of model calibration to observed prevaccination data are presented in **Supplementary Figures S1 and S2**.

### Sensitivity Analysis

Deterministic and probabilistic sensitivity analyses were performed to evaluate the robustness of the base case results when variations in the economic parameters and vaccine properties were applied. Economic variations included cost per vaccine dose (±20%) and treatment cost (±20% for direct and indirect costs). The impact of varying vaccine coverage (±5% for primary vaccination and booster vaccination) and primary vaccine take (-5% for V-MSD and ±5% for V-GSK; high [+5%] V-MSD primary take was not modeled as take cannot be >100%) were also assessed. Outcomes for the direct sensitivity analysis were incremental cost per QALY gained with respect to no vaccination. Outcomes for the probabilistic sensitivity analysis were plotted as scatterplots of incremental cost vs QALYs gained.

## RESULTS

### Clinical Varicella-Related Outcomes

**Figure 1.**
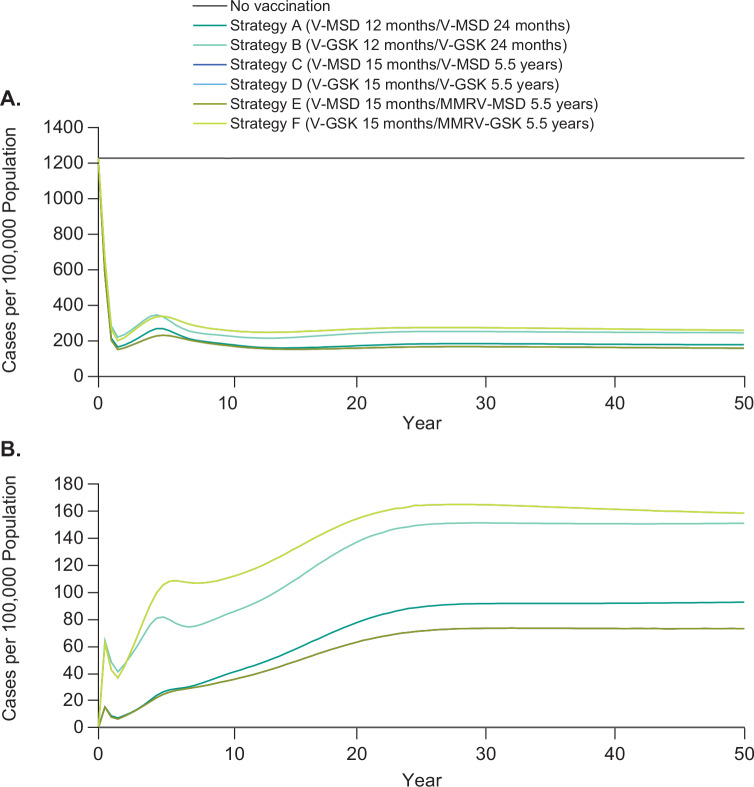
Total **(A)** and Breakthrough **(B)** Varicella Incidence After Implementation of UVV With Each Strategy Over 50 Years Strategies C and E and strategies D and F resulted in identical reductions in the incidence of total **(A)** and breakthrough **(B)** varicella. Therefore, the lines overlap in the graphs and cannot be distinguished. Abbreviations: GSK, GlaxoSmithKline Biologicals, Belgium, UK; MMRV, measles, mumps, rubella, and varicella vaccine; MSD, Merck & Co, Inc, Rahway, New Jersey; V, varicella vaccine; UVV, universal varicella vaccination.

In the base case, in the absence of UVV, there were an estimated 1228 varicella cases per 100 000 population over a 50-year period. Regardless of vaccination strategy, implementation of universal vaccination substantially reduced the incidence of total varicella cases to 159 to 260 per 100 000 population at 50 years (**Figure 1A**). The greatest reduction in varicella incidence was seen with strategy C (V-MSD 15 months/V-MSD 5.5 years) and strategy E (V-MSD 15 months/MMRV-MSD 5.5 years). After 50 years, the incidence of breakthrough varicella was lowest with strategies C and E (73 per 100 000 cases) and highest with strategies D and F (158 per 100 000 cases) (**Figure 1B**).

**Figure 2.**
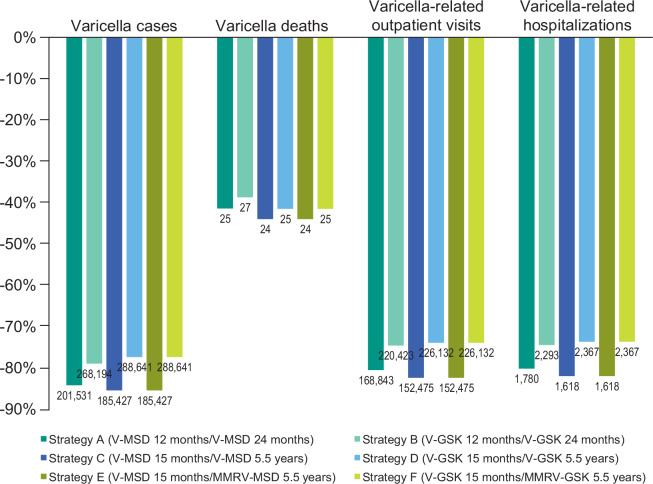
Percentage Change in Varicella-Related Clinical Outcomes vs No Vaccination, by Vaccination Strategy Bar labels indicate cumulative numbers over a 50-year period. Percent reductions indicate cumulative reductions in outcomes. Abbreviations: GSK, GlaxoSmithKline Biologicals, Belgium, UK; MMRV, measles, mumps, rubella, and varicella vaccine; MSD, Merck & Co, Inc, Rahway, New Jersey; V, varicella vaccine.

All 2-dose vaccination strategies were estimated to significantly reduce varicella cases (77%-85%), varicella deaths (39%-44%), outpatient cases (74%-82%), and varicella-related hospitalizations (74%-82%) at 50 years (**Figure 2**). The greatest reduction in varicella cases (85%), deaths (44%), outpatient cases (82%), and hospitalizations (82%) was observed for strategies C and E.

### Economic Outcomes

In the absence of UVV, total direct treatment costs for natural varicella exceeded €19.2 million over the 50-year time horizon, and indirect costs amounted to more than €165.6 million (**Table 2**). Introduction of UVV was projected to reduce varicella direct treatment cost, including for both natural and breakthrough varicella, to €3.2 million (strategies C and E) and €4.7 million (strategies D and F), and to reduce indirect costs to €28.3 million (strategies C and E) and €41.3 million (strategies D and F). Reductions in treatment costs and indirect costs were partially offset by the cost of vaccination, which ranged from €40.5 million for strategy C to €47.0 million for strategy E.

**Table 2.** Economic Impact of Implementing Vaccination Strategies vs No Vaccination at 50 Years, From Payer and Societal Perspectives

**Strategy**	**Total QALYs Per Capita**	**Total Cost Per Capita (**€)	**ΔQALY**	**ΔCost (**€)	**ICER (**€)
Payer perspective					
No vaccination	-0.001368	9.27			
Strategy A (V-MSD 12 mo/V-MSD 24 mo)	-0.000364	22.21	0.001004	12.94	12 892.57
Strategy B (V-GSK 12 mo/V-GSK 24 mo)	-0.000388	22.58	0.000980	13.31	13 587.41
Strategy C (V-MSD 15 mo/V-MSD 5.5 y)	-0.000355	21.02	0.001012	11.75	11 607.80
Strategy D (V-GSK 15 mo/V-GSK 5.5 y)	-0.000393	21.21	0.000975	11.94	12 246.17
Strategy E (V-MSD 15 mo/MMRV-MSD 5.5 y)	-0.000355	24.16	0.001012	14.89	14 708.59
Strategy F (V-GSK 15 mo/MMRV-GSK 5.5 y)	-0.000393	24.17	0.000975	14.90	15 284.46
Societal perspective					
No vaccination	-0.001368	88.94			
Strategy A (V-MSD 12 mo/V-MSD 24 mo)	-0.000364	36.94	0.001004	-52.01	Dominant
Strategy B (V-GSK 12 mo/V-GSK 24 mo)	-0.000388	41.46	0.000980	-47.48	Dominant
Strategy C (V-MSD 15 mo/V-MSD 5.5 y)	-0.000355	34.63	0.001012	-54.31	Dominant
Strategy D (V-GSK 15 mo/V-GSK 5.5 y)	-0.000393	41.09	0.000975	-47.85	Dominant
Strategy E (V-MSD 15 mo/MMRV-MSD 5.5 y)	-0.000355	37.77	0.001012	-51.17	Dominant
Strategy F (V-GSK 15 mo/MMRV-GSK 5.5 y)	-0.000393	44.06	0.000975	-44.89	Dominant

Abbreviations: GSK, GlaxoSmithKline Biologicals, Belgium, UK; ICER, incremental cost-effectiveness ratio; MMRV, measles, mumps, rubella, and varicella vaccine; MSD, Merck & Co, Inc, Rahway, New Jersey; QALY, quality-adjusted life-year. V, varicella vaccine.

Dominant: Strategy is both more effective (results in more QALYs) and less costly compared with no vaccination. Costs and QALYs were discounted at 3% annually.

**Cost-effectiveness of varicella vaccination—payer perspective**: In the absence of UVV, the total costs per capita were estimated to be €9.27 from the payer perspective. The 6 two-dose vaccination strategies considered resulted in total per capita costs ranging from €21.02 (strategy C) to €24.17 (strategy F), including costs for treatment and vaccination (**Table 2**). Strategies C and D were associated with the lowest incremental per capita costs of €11.75 and €11.94, respectively, suggesting that the 2-dose medium-interval strategies with the monovalent vaccinations V-MSD or V-GSK resulted in the lowest cost increase. In contrast, strategies E and F, representing medium-interval strategies with a quadrivalent vaccine at the second dose (MMRV-MSD or MMRV-GSK), resulted in the highest estimated incremental per capita costs at €14.89 and €14.90, respectively, reflecting the higher cost of quadrivalent vaccination doses.

The absence of UVV resulted in 0.00137 QALYs lost per capita. Strategies C and E were associated with the lowest number of QALYs lost (0.00036 QALYs for both), which represents a per capita gain of 0.00101 QALYs vs no vaccination. Strategies D and F were estimated to result in 0.00097 QALYs gained per capita compared with no vaccination, the lowest result among the 6 strategies considered.

From the payer perspective, all 6 vaccination strategies were cost-effective at the ICER threshold for Slovenia (€25000/QALY),^32^ with ICERs ranging from €11 608 per QALY (strategy C) to €15 284 per QALY (strategy F) compared with no vaccination. Strategy C dominated strategies A, B, D, E, and F as it achieved more QALYs for a lower cost. MSD strategies resulted in lower ICERs than the corresponding GSK strategies for all modeled vaccination schedules from the payer perspective (**Table 2**).

**Cost-effectiveness of varicella vaccination—societal perspective**: From the societal perspective, the total costs per capita were estimated to be €88.94 in the absence of UVV. All strategies resulted in reduced costs vs no vaccination, with total per capita costs ranging from €34.63 (strategy C) to €44.06 (strategy F) across the 6 strategies. The greatest reduction in total costs per capita was seen with the MSD strategies: strategy C (€54.31 reduction) followed by strategies A and E (€52.01 and €51.17).

From the societal perspective, all vaccination strategies were cost-saving compared with the no-vaccination strategy, as they provided greater QALYs at a lower cost (**Table 2**). Of the 6 strategies modeled, strategy C provided the greatest cost savings at the highest QALY gain and was dominant over the other strategies and no vaccination. As seen with the payer perspective, MSD strategies resulted in lower ICERs than the corresponding GSK strategies from the societal perspective as well.

### Direct Sensitivity Analysis

**Impact on ICER—payer perspective**: For all strategies, incremental cost per QALY gained for the payer perspective vs no vaccination was most sensitive to cost per vaccine dose (**Supplementary Figure S3**). Twenty percent variation in the cost per dose resulted in 26% to 28% variation in ICER for strategies A, B, C, and D. For strategies E and F, changes in quadrivalent and monovalent cost per dose resulted in variations of 15% and 13%, respectively. For all parameter ranges explored, ICER values remained below the cost-effectiveness threshold of €25 000 for Slovenia.

**Figure 3.**
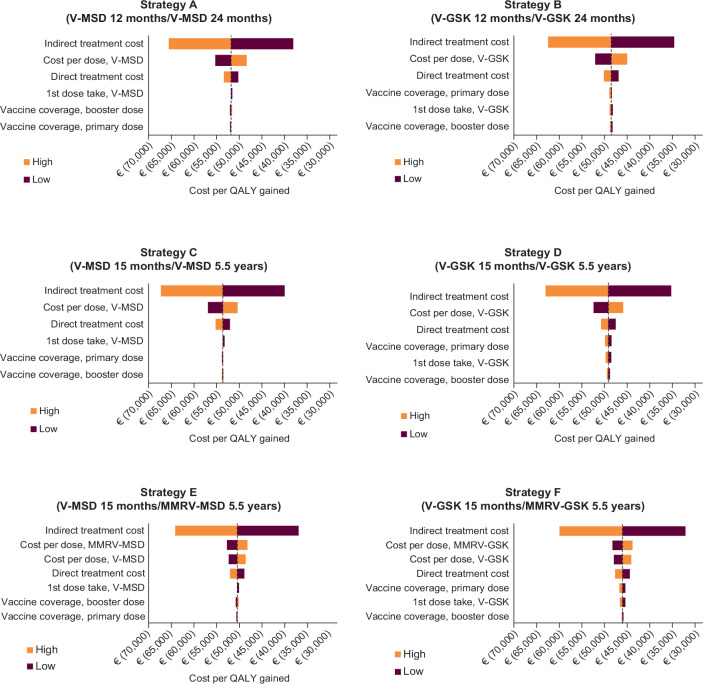
Change in ICER vs No Vaccination After Varying Economic Parameters and Vaccine Properties, From the Societal Perspective Cost parameters were varied by ±20%; vaccine coverage by ±5%; vaccine take was -5% for V-MSD, and ±5% for V-GSK. Abbreviations: GSK, GlaxoSmithKline Biologicals, Belgium, UK; MMRV, measles, mumps, rubella, and varicella vaccine; MSD, Merck & Co, Inc, Rahway, New Jersey; QALYs, quality-adjusted life-years; V, varicella vaccine.

**Impact on ICER—societal perspective**: From the societal perspective, indirect cost associated with workdays lost due to disease had the greatest impact on incremental cost per QALY gained for all strategies (**Figure 3**). Twenty percent variation in indirect cost resulted in 25% to 30% variation in ICER for all UVV strategies. For all parameter ranges explored, ICER values remained negative, pointing to societal cost savings associated with all vaccination strategies explored.

### Probabilistic Sensitivity Analysis

Results of the probabilistic sensitivity analysis are shown in **Supplementary Figures S4 and S5**. For the payer perspective for all UVV strategies, all data points lie below the cost-effectiveness threshold for Slovenia, reflecting the robustness of cost-effectiveness of varicella vaccination strategies to variation in economic and vaccine parameters. For the societal perspective (**Supplementary Figure S5**) for all UVV strategies, all data points have negative incremental costs for positive QALYs gained. This indicates that cost savings is maintained for varicella vaccination strategies from the societal perspective under variations in economic and vaccine parameters.

## DISCUSSION

In this study, all 2-dose UVV strategies modeled were predicted to reduce morbidity and mortality in Slovenia compared with no vaccination. At 50 years, varicella vaccination was estimated to reduce varicella cases by 77% to 85%, outpatient cases by 74% to 82%, hospitalizations by 74% to 82%, and varicella deaths by 39% to 44%. Given the current burden of varicella in the absence of universal vaccination in Slovenia,^6,8^ these results demonstrate the benefit of introducing a 2-dose universal vaccination schedule. All 6 strategies modeled would be cost-effective in Slovenia from the payer perspective based on the €25 000/QALY ICER threshold.^32^ From the societal perspective, all vaccination strategies were cost-saving compared with the no-vaccination strategy, providing greater health benefits at a lower cost. From both payer and societal perspectives, strategy C (V-MSD 15 months/V-MSD 5.5 years) was dominant over all strategies, resulting in the lowest cost and best improvement in QALYs.

The results of the current study support previous findings from dynamic transmission models of UVV for other European countries. Introducing a 2-dose strategy into national vaccination programs in Sweden reduced annual cases from 120 000 to less than 300 across all age groups and was cost-effective compared with no vaccination from the societal and payer perspectives.^37^ A significant reduction in cases was also predicted following the introduction of UVV in Turkey, where 2-dose vaccination was cost-effective.^23^ In Italy, the introduction of 2-dose UVV was predicted to have a major impact on reducing varicella incidence, prevalence, and societal costs, and all strategies assessed were cost-saving vs no vaccination.^38^ Similarly, in Switzerland, UVV was estimated to reduce the number of cases by 88% to 90%, hospitalizations by 62% to 69%, and deaths by 75% to 77% vs no vaccination or 10% private market use.^39^ Two-dose UVV was a cost-effective option compared with current recommendations and clinical practice in Switzerland, with significant reductions in the societal economic burden of disease.^39^ Finally, in Norway, 2-dose UVV was predicted to result in substantial reductions in outpatient cases, hospitalizations, and deaths, and was cost-effective vs no vaccination from payer and societal perspectives over the 50-year time horizon.^40^ Most of the projected cost savings from the societal perspective were due to reductions in lost workdays.^40^ In this study, all vaccination strategies were cost-saving compared with the no-vaccination strategy from the societal perspective, with indirect costs constituting a significant proportion of the societal costs associated with varicella. MSD strategies were more cost-effective than corresponding GSK strategies from the payer perspective, and more cost- saving from the societal perspective.

The model described here has been reviewed for accuracy and epidemiological appropriateness, and the results are consistent with previous model adaptations performed for Italy, Norway, and Turkey.^23,38,40^ In addition, results of this model are consistent with real-world observational studies showing the impact of UVV in countries such as Italy and the United States.^41–43^ Comparing the economic impact of vaccination between countries can be challenging given the heterogeneity in varicella infection reporting and differences in health care systems and vaccine pricing across countries. Nonetheless, the similarity of results between this modeling study and those previously reported highlights the financial benefit of varicella vaccination from both payer and societal perspectives.

The impact of varicella vaccination was also modeled in a previous study in Slovenia. Ahčan et al^44^ modeled 1-dose varicella vaccination (V-GSK) given at 15 months alongside MMR vaccination, assuming a 95% coverage rate and 90% vaccine efficacy. The authors reported a benefit-cost ratio of 0.89, concluding that there was no economic benefit of UVV in Slovenia.^44^ However, comparisons with the current study are limited, given the age of the data used in the previous model and differences in strategies assessed. While a single dose of varicella vaccine, as modeled by Ahčan et al,^44^ provides a good level of protection, previous studies have emphasized the additional benefit and heightened vaccine effectiveness of 2-dose vs 1-dose regimens. For example, in Germany, where routine vaccination was introduced in 2004, with the second dose recommended in 2009, the incremental vaccine effectiveness of adding a second dose was 68.9% (vaccine effectiveness of 1 vs 2 doses, 81.9% vs 94.4%).^45^ Although a cost-benefit ratio was not calculated, the present study found that all 2-dose strategies were cost-effective vs no vaccine at the ICER threshold for Slovenia. Further, this study also provides a more up-to-date picture of the health and economic impact of UVV on the current burden of disease in Slovenia.

Although benefits of varicella vaccination are well established in both randomized control trials and effectiveness studies, only 40 countries have implemented UVV in their national immunization programs globally. The most common reason for this is that varicella is generally considered a mild disease. However, our study and other published literature shows that varicella poses significant economic and clinical burdens. Hence, UVV could be a potential solution to reduce the burden of disease on both healthcare systems and society.

### Limitations

There were several limitations to this analysis. First, the ICER results were conservative estimates, as the analysis used the list price instead of the tender price for all vaccines modeled. Price parity was also assumed between MSD and GSK vaccines. Second, a static population for Slovenia was assumed for the analysis; however, more realistic demographic assumptions may change transmission patterns, especially among older individuals, which could impact health and economic outcomes. Third, proxy estimates from other countries were used if country specific inputs were not available; therefore, the data may not be fully representative of Slovenia. Fourth, there is currently no recommended healthcare visit at 24 months in Slovenia; the model did not consider the additional cost of a new visit, only the additional vaccination administration cost. Lastly, the impact of UVV on herpes zoster incidence was not assessed in outcomes.

## CONCLUSIONS

In conclusion, 2-dose UVV was projected to substantially reduce the morbidity and mortality burden of varicella in Slovenia compared with no vaccination over a 50-year time horizon. All 2-dose UVV strategies were cost-effective relative to no vaccination from the payer perspective, and cost-saving compared with no vaccination from the societal perspective, regardless of product or dosing interval (short or medium). While all strategies modeled were predicted to be favorable vs no vaccination in Slovenia, the most cost-effective strategy from the payer and societal perspective included V-MSD for both doses (15 months/5.5 years), resulting in the largest cost savings and greatest health improvements. Policymakers should consider a UVV strategy to reduce the burden of disease in Slovenia.

### Author Contributions

C.B. participated in the design of the study, acquisition and analysis of the data, interpretation of the results, and drafting of the manuscript; S.A.K. participated in the conception, design and planning of the study, acquisition of the data, interpretation of the results, and drafting of the manuscript; A.L. participated in the acquisition of the data, interpretation of the results, and drafting and critically reviewing the manuscript; G.B. participated in analysis of the data, interpretation of the results, and critically reviewing the manuscript; M.P. participated in the conception, design, and planning of the study, analysis of the data, interpretation of the results, and drafting and critically reviewing the manuscript.

### Disclosures

M.P. and S.A.K. are employees of Merck Sharp & Dohme Corp, a subsidiary of Merck & Co, Inc, Rahway, New Jersey, and may hold stock or stock options in Merck & Co, Inc, Rahway, New Jersey. C.B. is a contractor with Merck Sharp & Dohme Corp, a subsidiary of Merck & Co, Inc, Rahway, New Jersey. G.B. is an employee of MSD Spain and may hold stock or stock options in Merck & Co, Inc, Rahway, New Jersey. A.L. is an employee of MSD Inovativna zdravilla and may hold stock or stock options in Merck & Co, Inc, Rahway, New Jersey.

### Meeting Presentation

Initial findings from this study were presented at the European Society of Pediatric Infectious Diseases (ESPID) Virtual Meeting 2021 in ePoster format, and the abstract is included in the ESPID 2021 abstract book (available online).

## Supplementary Material

Online Supplementary Material
