# Supplementary material for: The Long-Term Clinical and Economic Impact of Universal Varicella Vaccination in Slovenia
Source: J Health Econ Outcomes Res. 2022 Sep 20;9(2):95–102. doi: 10.36469/001c.37308 (PMC9489276; doi:10.36469/001c.37308)
Supplement: Online Supplementary Material [file jheor_2022_9_2_37308_100284.pdf]

Journal of Health Economics and Outcomes Research

Online Supplementary Material

The Long-term Clinical and Economic Impact of Universal Varicella Vaccination in Slovenia. *JHEOR*. 2022;9(2):95-102. doi:10.36469/jheor.2022.37308

Table S1: Vaccine Characteristics for Modeled Vaccine Formulations

Table S2: Base Model Parameters (Disease-Specific)

Table S3: Key Model Inputs and Data Source for Varicella Infection in Slovenia

Table S4: Health State Utility Values Used in the Model

Figure S1: Model Calibration to Observed Prevaccination Varicella Seroprevalence by Age in Slovenia

Figure S2: Model Calibration to Observed Prevaccination Herpes Zoster Incidence by Age in Slovenia

Figure S3: Change in ICER vs No Vaccination After Varying Economic Parameters and Vaccine Properties, From the Payer Perspective

Figure S4: Results of Probabilistic Sensitivity Analysis Varying Economic Parameters and Vaccine Properties, From the Payer Perspective

Figure S5: Results of Probabilistic Sensitivity Analysis Varying Economic Parameters and Vaccine Properties, From the Societal Perspective

This supplementary material has been provided by the authors to give readers additional information about their work.

This is an open-access article distributed under the terms of the Creative Commons Attribution 4.0 International License (CCBY-4.0). View this license's legal deed at http://creativecommons.org/licenses/by/4.0 and legal code at http://creativecommons.org/licenses/by/4.0/legalcode for more information.

Table S1. Vaccine Characteristics for Modeled Vaccine Formulations

Vaccine/Dose	Failure (%)	Vaccine Take, Range (%)	Duration of Protection, Range (y)	References
First dose				
MSD varicella-containing vaccines	4	100 (93-100)	25 (15-67)	Kuter et al (2004)[1]; van Hoek et al (2011)[2]
GSK varicella-containing vaccines	5	65.4 (57.2-72.1)	17 (NR)	Holl et al (2016)[3]; Kuter et al (2004)[1]; Ouwens et al (2015)[4]; Prymula et al (2014)[5]; van Hoek et al (2011)[2]
Second dose				
MSD varicella-containing vaccines	NA	100 (97-100)	77 (38-200)	Kuter et al (2004)[1]; van Hoek et al (2011)[2]
GSK varicella-containing vaccines	NA	94.9 (92.4-96.6)	77 (NR)	Holl et al (2016)[3]; Ouwens et al (2015)[4]; Prymula et al (2014)[5]

Abbreviations: GSK, GlaxoSmithKline Biologicals, Belgium, UK; MSD, Merck & Co, Inc, Rahway, New Jersey; NA, not applicable; NR, not reported.

Table S2. Base Model Parameters (Disease-Specific)

Symbol	Description	Value	Source
$1/\omega_m$	Average period of passive immunity	6 months	Gershon et al (1976)[6]
$1/\varepsilon_n$, $1/\varepsilon_{vb}$	Average latent period (natural, breakthrough varicella)	14 days	Marin & Bialek (2015)[7]; Brisson et al (2000)[8]
$1/\gamma_n$	Average infectious period for natural varicella	7 days	Bernstein et al (1993)[9]; Marin & Bialek (2015)[7]
$1/\gamma_{bv}$	Average infectious period for breakthrough varicella	4.5 days	Izurieta et al (1997)[10]
ρ_v	Relative infectivity of breakthrough varicella infection	50%	Seward et al (2004)[11]; Brisson et al (2000)[8]
ξ_n, ξ_{vv}, ξ_{vb}	Rate of endogenous boosting	0	Assumed
χ	Reactivation rate factor on vaccine arms	1/6	Poletti et al (2013)[12]
β	Transmission matrix		From calibration
μ	Force of mortality		Derived
d_v	Case fatality rate for natural varicella (per 100 000)	<1 y: 1.04	Wolfson et al (2019)[13]
		1-5 y: 0.26	
		5-10 y: 0.65	
		10-15 y: 3.12	
		15-45 y: 31.22	
		45-65 y: 322.56	
		≥65 y: 1461.06	

Table S3. Key Model Inputs and Data Source for Varicella Infection in Slovenia

Parameter	Values	Source
**Vaccination Costs**		
Vaccine[a,b] (€)	Varicella: 31.84	Centralna baza zdravil[14]; Österreichischer Apotheker-Verlag[15]; Uradni List Republike Slovenije, 2020[16]
	MMRV: 51.52	
Vaccine administration[c] (€)	5.62	Zavod Za Zdravstveno Zavarovanje Slovenije[17]
**Direct Costs**		
Percentage of cases requiring outpatient visits	0-5 y: 69%	Dozsa et al (2017)[18]
	5–9 y: 57%	
	≥10 y: 100%	
Cost per outpatient visit (€)	<15 y: 8.83	Zavod Za Zdravstveno Zavarovanje Slovenije[17]
	≥15 y: 14.28	
Percentage of cases requiring hospitalization	<15 y: 0.69	Ahčan et al. 2002[19]; Heininger & Seward (2006)[20]
	≥15 y: 1.38	
Cost per hospitalization (€)	672.78	Zavod Za Zdravstveno Zavarovanje Slovenije[17]
**Indirect Costs**		
Days lost from work (outpatient and inpatient)	<18 y: 5.5	Statistical office of the National Institute of Public Health[21]
	18-65 y: 12.3	
	65+ y: 0	
Cost per workday lost (€)	64.06	Republika Slovenija Statistični Urad[22]

[a]Varilrix® is not currently available in Slovenia, therefore, price parity with Varivax® was assumed.
[b]MMRV prices have been adjusted to reflect the V-component of the vaccine only.
[c]Vaccine administration costs include immunization, exam, organization, vaccination schedule, vaccine purchase, editing records, and reports. No additional administration costs are assumed for MMRV at the second dose.

Table S4. Health State Utility Values Used in the Model

Health State	Utility by Age (y)							
	**<18**	**18-25**	**25-35**	**35-45**	**45-55**	**55-65**	**65-75**	**75+**
Healthy (q_h)[23]	0.869	0.869	0.841	0.794	0.712	0.619	0.554	0.498
	**Utility by Age (y)**							
	**<15**	**15-40**	**40-60**	**60-70**	**70-80**		**80+**	
Natural varicella (q_n)[24]	0.79	0.74	0.74	0.74	0.74		0.74	
Breakthrough varicella (q_{vv})[24]	0.92	0.92	0.92	0.92	0.92		0.92	

Figure S1. Model Calibration to Observed Prevaccination Varicella Seroprevalence by Age in Slovenia

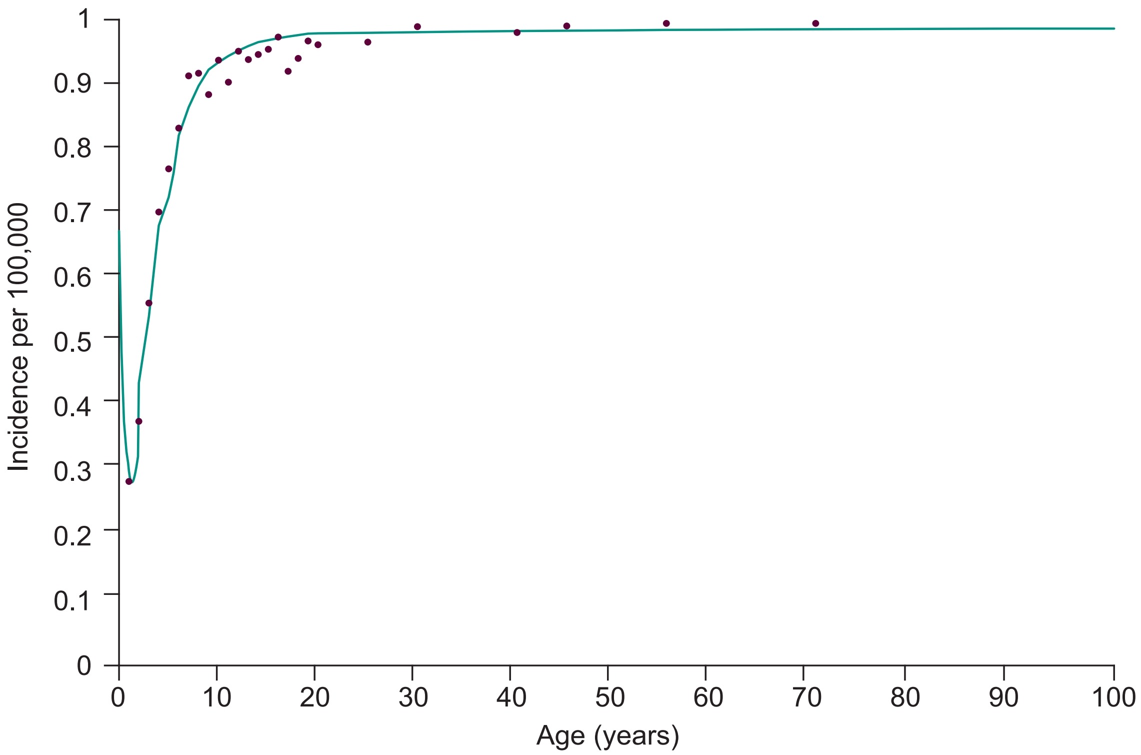

Figure S2. Model Calibration to Observed Prevaccination Herpes Zoster Incidence by Age in Slovenia

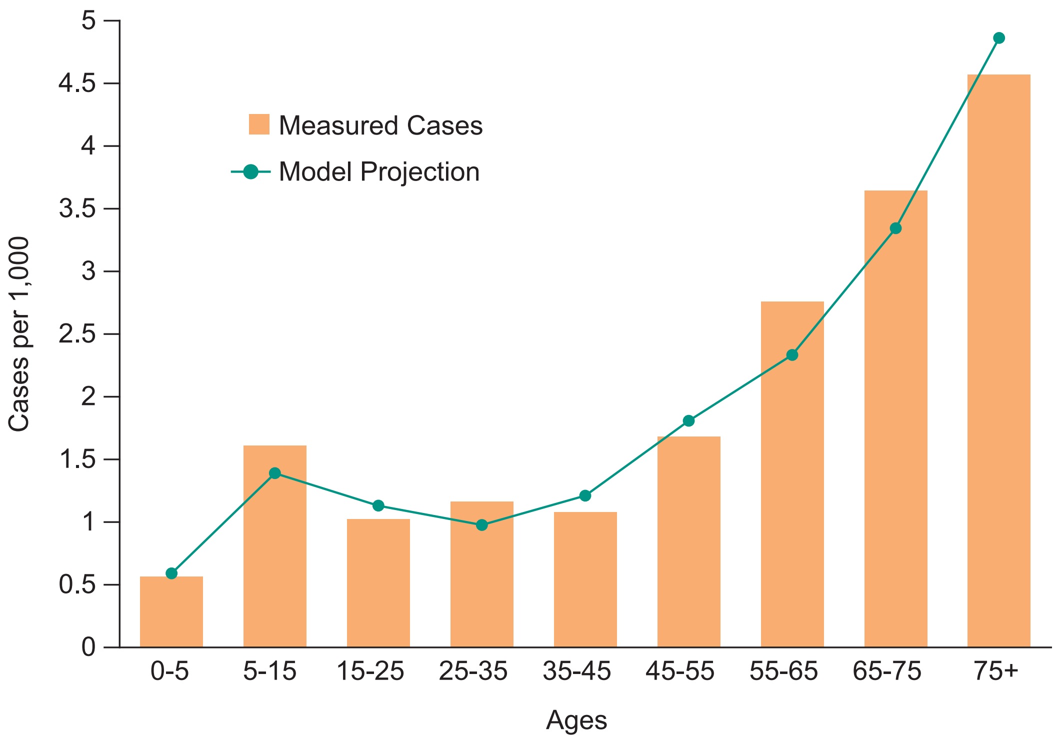

Figure S3. Change in ICER vs No Vaccination After Varying Economic Parameters and Vaccine Properties, From the Payer Perspective

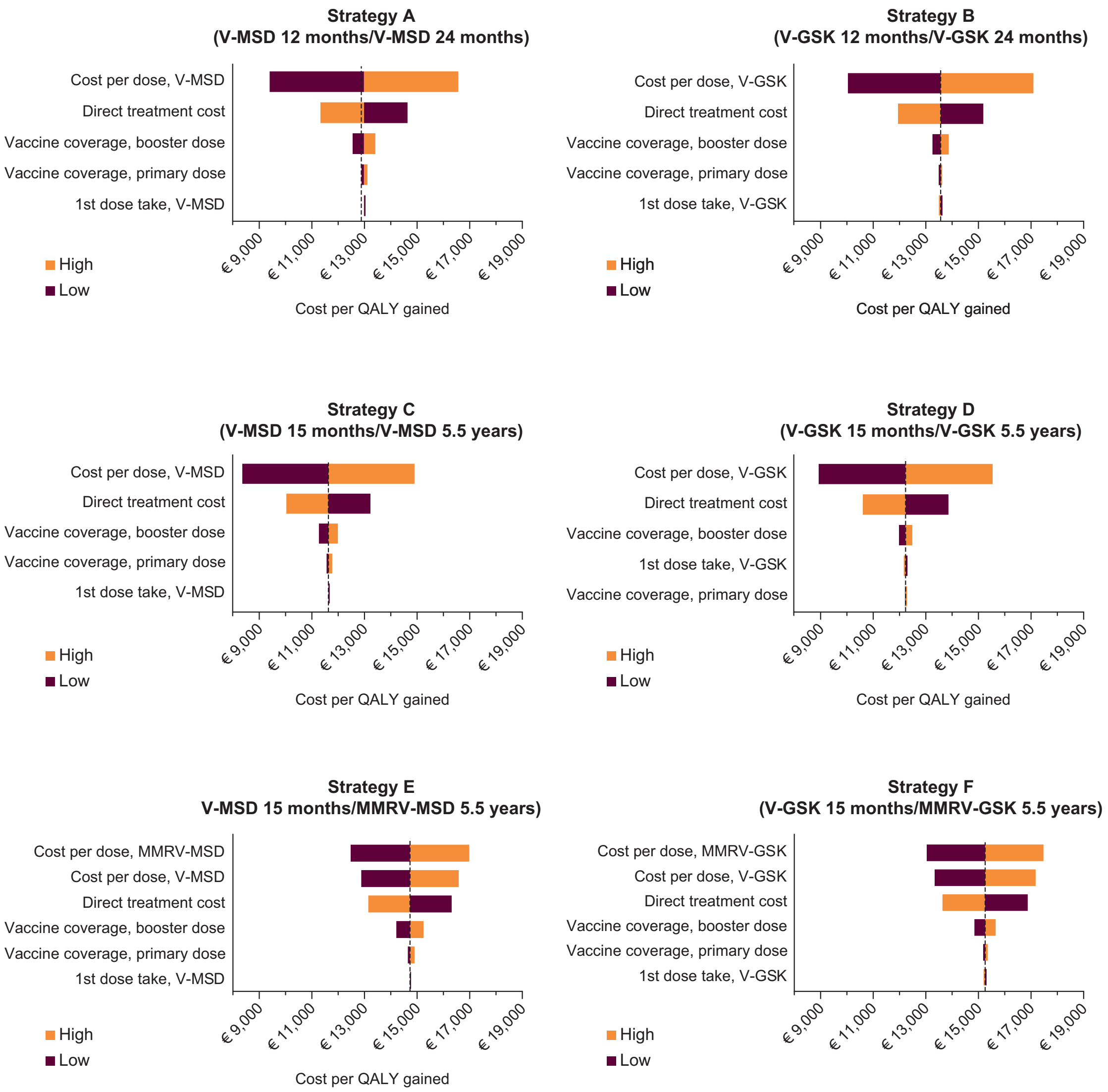

Abbreviations: GSK, GlaxoSmithKline Biologicals, Belgium, UK; MMRV, measles, mumps, rubella, and varicella vaccine; MSD, Merck & Co, Inc, Rahway, New Jersey; QALY, quality-adjusted life-year; V, varicella vaccine.

Figure S4. Results of Probabilistic Sensitivity Analysis Varying Economic Parameters and Vaccine Properties, From the Payer Perspective

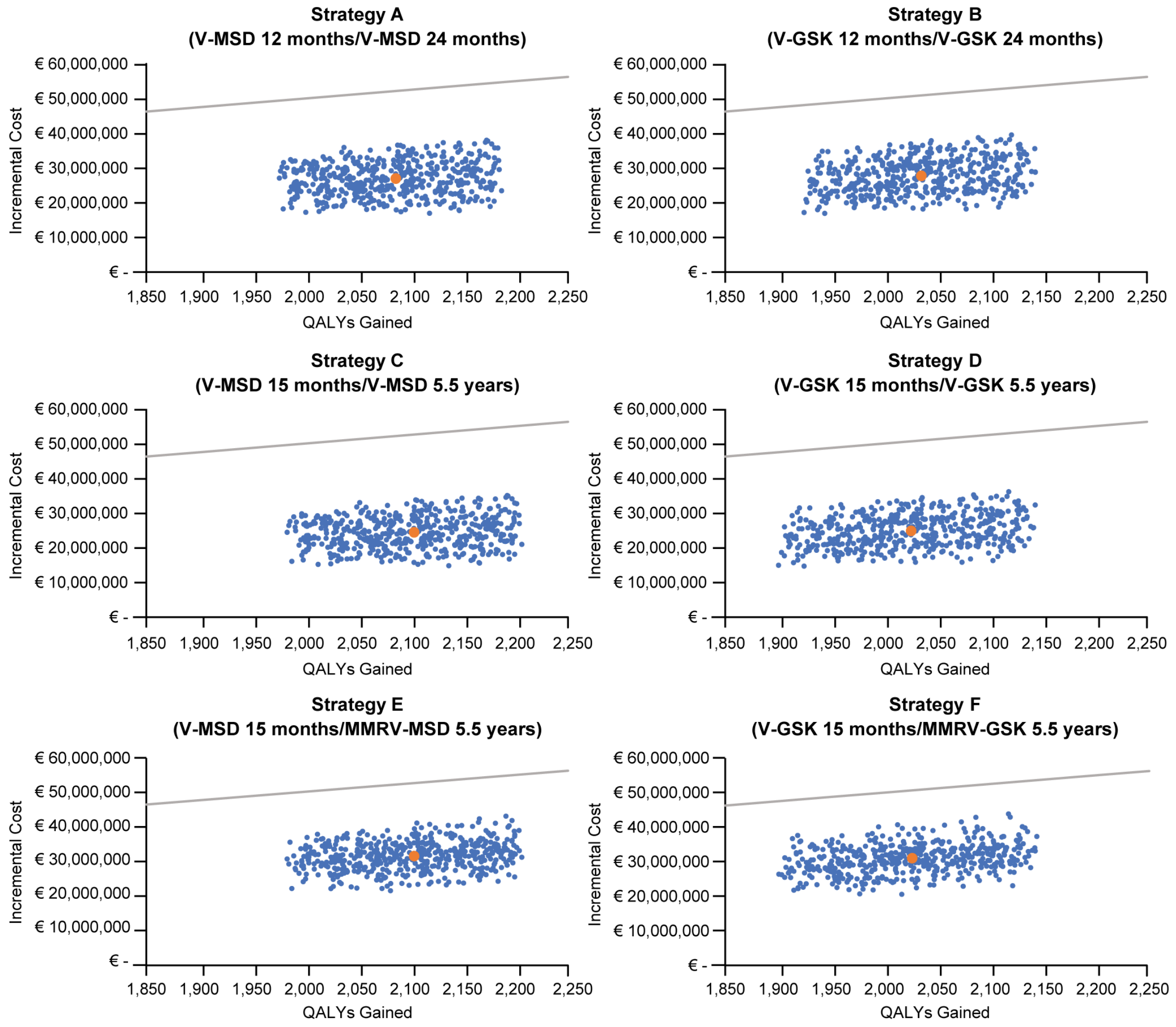

Solid gray line represents the cost-effectiveness threshold of €25 000 per QALY gained for Slovenia.
Abbreviations: GSK, GlaxoSmithKline Biologicals, Belgium, UK; MMRV, measles, mumps, rubella, and varicella vaccine; MSD, Merck & Co, Inc, Rahway, New Jersey; QALYs, quality-adjusted life-years; V, varicella vaccine.

Figure S5. Results of Probabilistic Sensitivity Analysis Varying Economic Parameters and Vaccine Properties, From the Societal Perspective

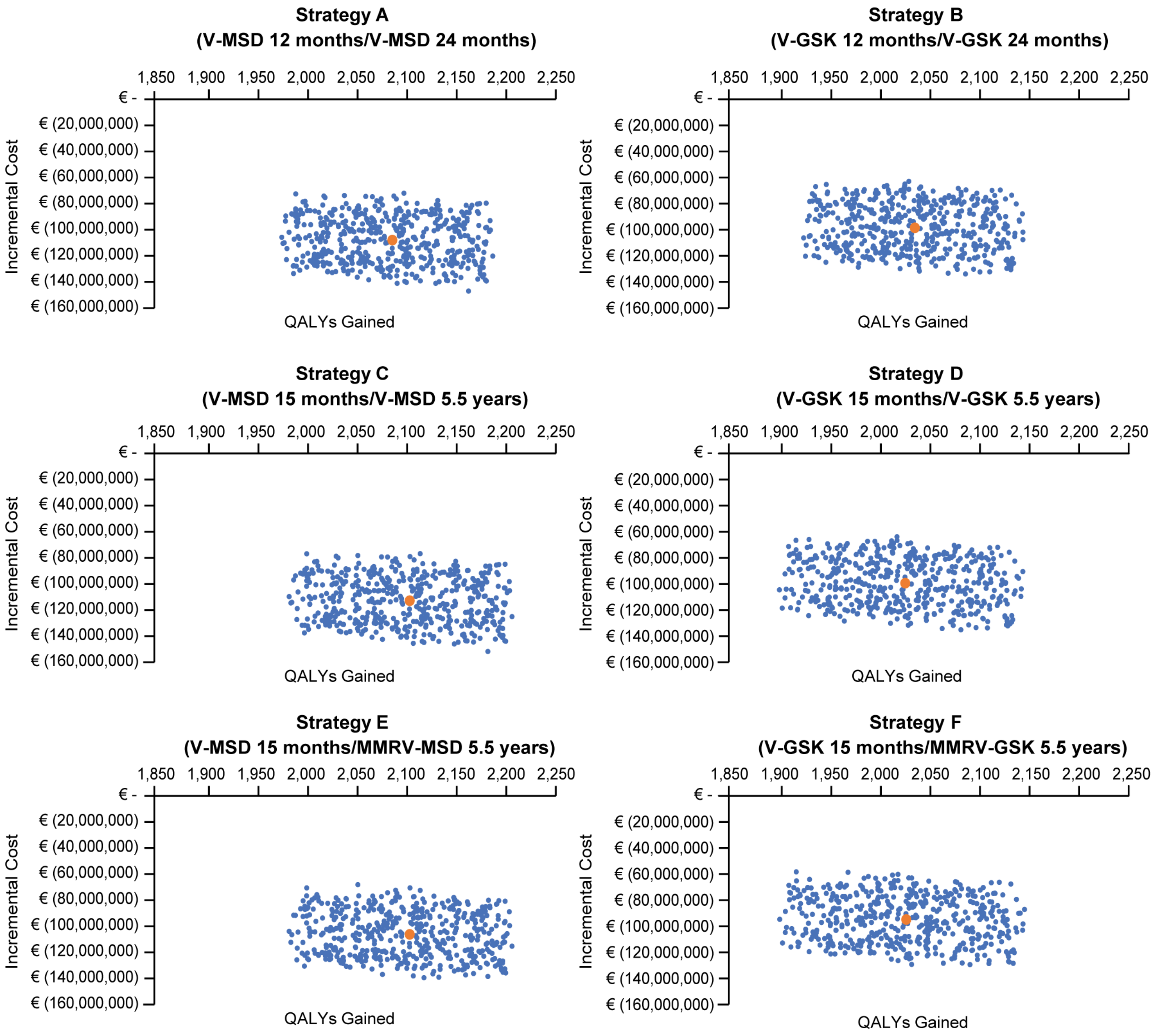

Abbreviations: GSK, GlaxoSmithKline Biologicals, Belgium, UK; MMRV, measles, mumps, rubella, and varicella vaccine; MSD, Merck & Co, Inc, Rahway, New Jersey; QALYs, quality-adjusted life-years; V, varicella vaccine.

REFERENCES

1. Kuter B, Matthews H, Shinefield H, et al. Ten year follow-up of healthy children who received one or two injections of varicella vaccine. *Pediatr Infect Dis J*. 2004;23(2):132-137.

2. Van Hoek AJ, Melegaro A, Zagheni E, Edmunds WJ, Gay N. Modelling the impact of a combined varicella and zoster vaccination programme on the epidemiology of varicella zoster virus in England. *Vaccine*. 2011;29(13):2411-2420.

3. Holl K, Sauboin C, Amodio E, Bonanni P, Gabutti G. Coverage, efficacy or dosing interval: which factor predominantly influences the impact of routine childhood vaccination for the prevention of varicella? A model-based study for Italy. *BMC Public Health*. 2016;16(1):1-11.

4. Ouwens MJ, Littlewood KJ, Sauboin C, et al. The impact of 2-dose routine measles, mumps, rubella, and varicella vaccination in France on the epidemiology of varicella and zoster using a dynamic model with an empirical contact matrix. *Clin Ther*. 2015;37(4):816-829. e810.

5. Prymula R, Bergsaker MR, Esposito S, et al. Protection against varicella with two doses of combined measles-mumps-rubella-varicella vaccine versus one dose of monovalent varicella vaccine: a multicentre, observer-blind, randomised, controlled trial. *Lancet*. 2014;383(9925):1313-1324.

6. Gershon AA, Raker R, Steinberg S, Topf-Olstein B, Drusin LM. Antibody to varicella-zoster virus in parturient women and their offspring during the first year of life. *Pediatrics*. 1976;58(5):692-696.

7. Marin M, Bialek SR. Varicella/Herpes Zoster. In: *Control of Communicable Diseases Manual*. 16th ed. Washington, DC: American Public Health Association; 2015.

8. Brisson M, Edmunds W, Gay N, Law B, De Serres G. Modelling the impact of immunization on the epidemiology of varicella zoster virus. *Epidemiol Infect.* 2000;125(3):651-669.

9. Bernstein HH, Rothstein EP, Watson BM, et al. Clinical survey of natural varicella compared with breakthrough varicella after immunization with live attenuated Oka/Merck varicella vaccine. *Pediatrics*. 1993;92(6):833-837.

10. Izurieta HS, Strebel PM, Blake PA. Postlicensure effectiveness of varicella vaccine during an outbreak in a child care center. *JAMA*. 1997;278(18):1495-1499.

11. Seward JF, Zhang JX, Maupin TJ, Mascola L, Jumaan AO. Contagiousness of varicella in vaccinated cases: a household contact study. *JAMA*. 2004;292(6):704-708.

12. Poletti P, Melegaro A, Ajelli M, et al. Perspectives on the impact of varicella immunization on herpes zoster. A model-based evaluation from three European countries. *PLoS One.* 2013;8(4):e60732.

13. Wolfson LJ, Daniels VJ, Pillsbury M, et al. Cost-effectiveness analysis of universal varicella vaccination in Turkey using a dynamic transmission model. *PLoS One*. 2019;14(8):e0220921.

14. Centralna baza zdravil. Podrobni podatki o zdravilu - Varivax; Nacionalna šifra zdravila: 041599. 2019. Accessed November 2021. http://www.cbz.si/cbz/bazazdr2.nsf/o/94A96582A326CDEBC12579C2003F64AC?opendocument

15. Österreichischer Apotheker-Verlag. Waren Verzeichnis. Accessed November 2021. https://warenverzeichnis.apoverlag.at/

16. Uradni List Republike Slovenije. Državni Zbor 16 March 2020. Accessed November 2021. https://www.uradni-list.si/_pdf/2020/Ur/u2020026.pdf

17. Zavod Za Zdravstveno Zavarovanje Slovenije. Akontativne cene 2020. Published 2020 (SD 2018). Accessed December 2019. http://www.zzzs.si

18. Dozsa C, Mezner, Z., Patri, L., Benedek, A., Kalmar, J., Weiss, T.J., et al. The costs and benefits of varicella vaccination in Hungary. Paper presented at: World Congress of the Society for Pediatric Infectious Disease (WSPID): 10th conference. December 2-5, 2017; Shenzen, China.

19. Ahčan J, Cizman M, Pleterski-Rigler D, Rakar R. Value of universal childhood varicella vaccination in Slovenia. *Zdr Vestn.* 2002;71(11):667-672.

20. Heininger U, Seward JF. Varicella. *Lancet.* 2006;368(9544):1365-1376.

21. Statistical office of the National Institute of Public Health. Data for 2014–2018. Email communication; April 2020.

22. Republika Slovenija Statistični Urad. Plače in stroški dela. Accessed November 2021. https://www.stat.si/StatWeb/Field/Index/15

23. Szende A, Janssen B, Cabases J. Self-Reported Population Health: An International Perspective based on EQ-5D. Dordrecht (NL): Springer; 2014.

24. Brisson M, Edmunds WJ. Varicella vaccination in England and Wales: cost-utility analysis. *Arch Dis Child*. 2003;88(10):862-869.
